# Chemokines in the resolution of inflammation: key players and targets for therapeutic modulation

**DOI:** 10.3389/fimmu.2025.1717666

**Published:** 2025-11-19

**Authors:** Vivian Louise Soares Oliveira, Paul Proost, Sofie Struyf

**Affiliations:** Laboratory of Molecular Immunology, Department of Microbiology, Immunology and Transplantation, Rega Institute, KU Leuven, Leuven, Belgium

**Keywords:** chemokines, chemokine receptors, resolution of inflammation, immune modulation, leukocyte recruitment

## Abstract

The resolution of inflammation is an active, tightly regulated process essential for restoring tissue homeostasis after an inflammatory process. While chemokines are classically recognized for their roles in leukocyte recruitment and immune cell positioning during the onset of inflammation, emerging evidence highlights their pivotal functions in orchestrating the resolution phase, as well. The chemokine system contributes to inflammation resolution through several complementary mechanisms, including the depletion of pro-inflammatory chemokines, the generation of autoantibodies, the promotion of neutrophil reverse migration, the recruitment and polarization of pro-resolving immune cells such as macrophages and regulatory T cells, and the induction of tissue repair and disease recovery. Modulating chemokine-receptor interactions, enhancing the activity of pro-resolving chemokines, or blocking detrimental chemokine signaling pathways represent promising strategies for the treatment of excessive inflammation or chronic inflammatory diseases. In addition, modulation of glycosaminoglycan interactions or chemokine-modifying enzymes, might also be useful in this context. In this review, we explore the roles of chemokines in resolution, with a focus on their mechanistic contributions to immune modulation and their potential as therapeutic targets for restoring immune balance.

## Introduction

### Resolution of inflammation

Resolution of inflammation is a highly coordinated process that restores tissue homeostasis after an inflammatory response. This process is not a passive stop of inflammation but involves a dynamic shift in cellular and molecular mechanisms to counterbalance the pro-inflammatory phase (1). Effective resolution requires the termination of inflammatory signaling, the removal of pro-inflammatory immune cells and debris, and the initiation of tissue repair pathways. Failure in the resolution of inflammation can lead to chronic inflammation, fibrosis, and/or autoimmunity (2). One of the first steps in resolution is the decrease in production of pro-inflammatory mediators, including cytokines such as tumor necrosis factor alpha (TNF-α), interleukin (IL)-1β, and IL-6, as well as lipid mediators such as leukotrienes and prostaglandins. Simultaneously, neutrophil recruitment is ceased. Those neutrophils already present in inflamed tissues either go into apoptosis or may acquire anti-inflammatory properties, due to the production of vesicles that suppress complement activation, decreasing further neutrophil recruitment and reducing tissue damage (3). Eventually, apoptotic neutrophils in the tissue are cleared by macrophages in a process called efferocytosis, which not only removes cellular debris but also actively promotes the transition to a pro-resolving environment by triggering macrophage reprogramming toward an anti-inflammatory and tissue-repair phenotype (4–7). The subsequent cascade of pro-resolving responses involves a coordinated network of signaling events that collectively suppress inflammation and restore tissue homeostasis. Upon engulfing apoptotic neutrophils, macrophages undergo metabolic and transcriptional reprogramming characterized by increased production of anti-inflammatory cytokines such as IL-10 and transforming growth factor beta (TGF-β), as well as the downregulation of pro-inflammatory mediators (8, 9). This reprogramming not only limits further leukocyte recruitment but also fosters tissue regeneration by stimulating fibroblast activation, angiogenesis, and matrix remodeling (10, 11). In this way, the clearance of apoptotic neutrophils serves as a central turning point that transforms the inflammatory environment into one conducive to resolution and repair.

Among the cells involved in the resolution of inflammation, macrophages play an essential and multifaceted role. They originate from two major ontogenic sources: tissue-resident macrophages, arising from embryonic hematopoietic progenitors, and monocyte-derived macrophages, which develop from circulating monocytes during inflammation. This distinction is crucial, as tissue-resident macrophages are generally anti-inflammatory, maintaining homeostasis by constantly clearing apoptotic cells, which imprints a tolerogenic and anti-inflammatory phenotype (12, 13). In contrast, monocyte-derived macrophages are typically pro-inflammatory during the acute phase of inflammation, but can transition toward a pro-resolving phenotype as the inflammatory response subsides (14).

Besides the already mentioned efferocytosis, macrophages contribute to the production of pro-resolving mediators, including specialized pro-resolving lipid mediators (SPMs) such as resolvins, maresins, and protectins. Their synthesis can be stimulated by apoptotic bodies, extracellular vesicles from the microenvironment or through trans-cellular biosynthesis in cooperation with other cells. These mediators collectively facilitate the resolution of inflammation and promote tissue repair (4, 15–18). Additionally, by responding to signals from the environment, macrophages undergo polarization toward an anti-inflammatory and pro-resolving phenotype, commonly categorized as M2 macrophages (9, 19–21). A key feature of M2 macrophages is their expression of scavenger receptors such as CD163, CD206, and MerTK, which enhance their ability to clear apoptotic cells and diminish inflammation (4–6). Although the M1/M2 classification is widely used, it presents clear limitations. Transcriptomic analyses of murine macrophages revealed that macrophages associated with resolution exhibit a distinct genetic signature compared to conventional M2 macrophages. For instance, ccl5 transcripts are enriched, suggesting a specific role for this chemokine in the resolution process. This study also highlighted the differences between the cells polarized *in vitro* with defined cytokine stimuli and *in situ* macrophages (22). Thus, the proposed subdivision of M2 macrophages into additional subtypes remains controversial, and studies examining chemokines and their receptors in the context of macrophage polarization and resolution often fail to differentiate among various pro-resolving populations (23, 24). Therefore, we chose to maintain the simplified M1/M2 nomenclature, as further subdivision into M2 subtypes could introduce unnecessary complexity and potential confusion.

Besides macrophages, other cell types contribute to resolution of inflammation. Regulatory T cells (Tregs) secrete IL-10 and TGF-β, further dampening inflammation and supporting tissue repair. Innate lymphoid cells (ILCs), B cells and mesenchymal stromal cells also release pro-resolving mediators that modulate immune responses and prevent chronic inflammation (25–27). Additionally, endothelial cells and fibroblasts play a role in restoring vascular integrity and extracellular matrix homeostasis.

In summary, resolution of inflammation is a well-orchestrated process involving not only the cessation of pro-inflammatory responses but also the active engagement of cellular and molecular pathways that promote healing. The balance between pro-inflammatory and pro-resolving signals is crucial, making the study of resolution mechanisms highly relevant for the development of therapeutic strategies for treatment of inflammatory and autoimmune diseases.

### Chemokine system

Chemokines, or chemotactic cytokines, are a family of relatively small secreted proteins that signal through G protein-coupled receptors (GPCRs) and are structurally characterized by the presence of four conserved cysteine residues (28, 29). The hallmark function of chemokines is to induce and guide the directional movement of cells, especially leukocytes. Additionally, chemokines trigger the activation of integrins and initiate effector functions in the target cells, e.g., the release of granules by polymorphonuclear cells, or influence cell survival (30). Therefore, chemokines are important in inflammation, but specific chemokines also act in homeostasis, angiogenesis, and embryogenesis (31–33). Based on the position of the cysteine residues in the N-terminal region of their sequence, chemokines are classified into 4 subfamilies (1): CC chemokines have two adjacent N-terminal cysteines (2), CXC chemokines present one amino acid between the two first cysteines (3), the CX3C chemokine has 3 amino acids between the two first cysteines, and (4) C chemokines possess only one of the usually conserved N-terminal cysteines. The CXC chemokine subfamily can be further subclassified into Glu-Leu-Arg (ELR)+ and ELR- CXC chemokines. The ELR+ CXC chemokines are associated with neutrophil recruitment, signal through chemokine receptors CXCR1 and/or CXCR2 and in humans include CXCL1, CXCL2, CXCL3, CXCL5, CXCL6, CXCL7, and CXCL8 (34, 35).

#### Chemokine receptors

In addition to GPCRs, chemokines can bind to atypical chemokine receptors (ACKR) (36) and glycosaminoglycans (GAGs) (37). Chemokine-binding GPCRs are classified as CCR, CXCR, CX3CR, and XCR according to the cysteine motif in their ligands (38, 39). Interestingly, one chemokine sometimes binds different receptors and one receptor may transduce signals upon engagement of distinct ligands. These interactions elucidate apparent bias in the chemokine system, which allows us to understand how one chemokine might promote different responses in different contexts (40, 41). A GPCR consists of a single polypeptide chain that is folded into a globular shape and anchored in the cell’s membrane. These receptors possess seven transmembrane helices, three extra-cellular and three intra-cellular loops. Chemokines typically interact with two interaction sites on their receptors. One being the extracellular N-terminal part of the receptor and a second in a pocket more buried inside the receptor. The extracellular loops together with residues in the transmembrane domains form these chemokine binding pockets. Binding of chemokines in those pockets induces intracellular signaling by second messengers such as calcium, cyclic adenosine monophosphate, and GTPases (42, 43).

**Figure 1:**
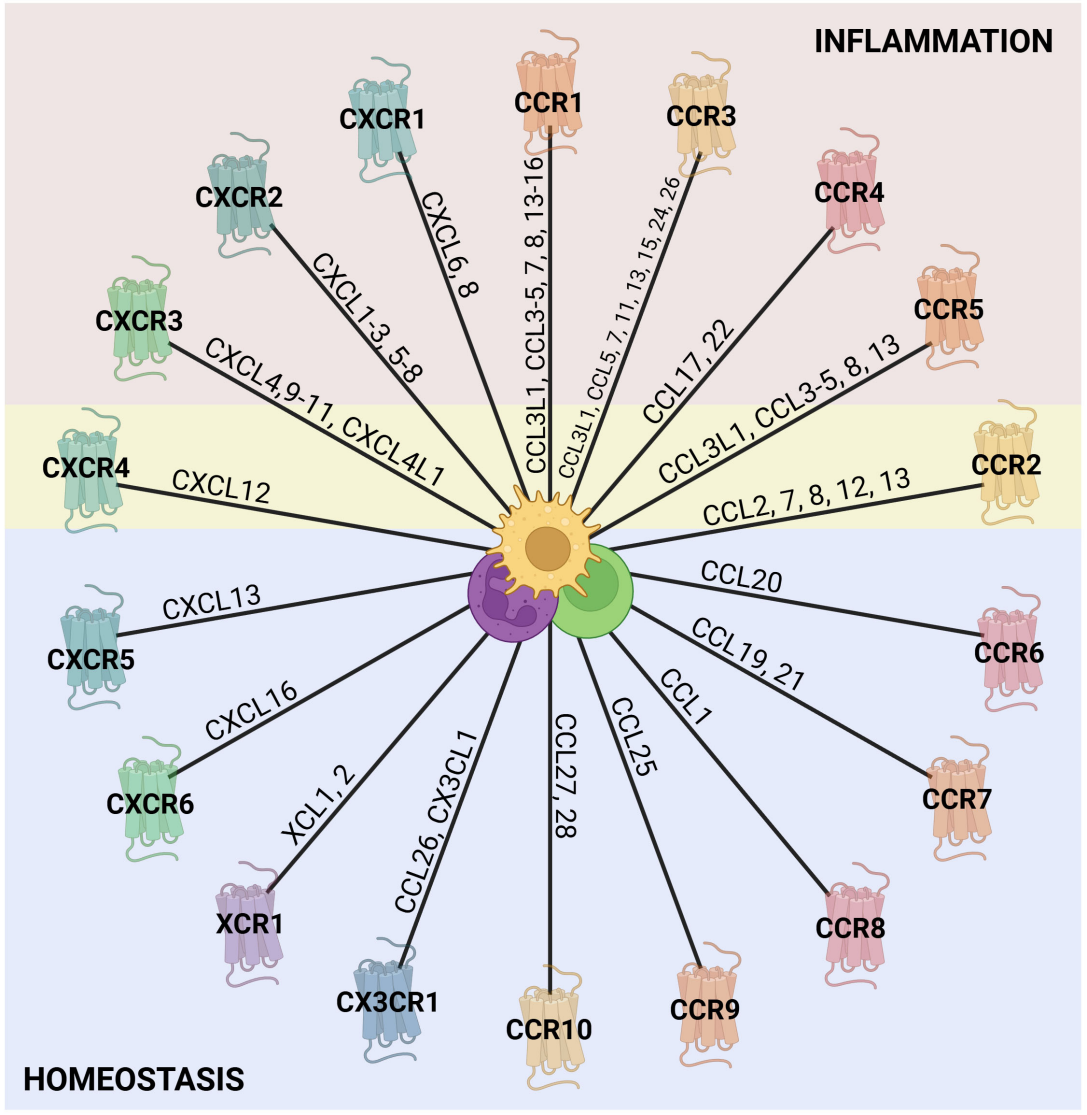
Chemokine receptors and their ligands. The outer ring of the wheel is composed of representations of the known chemokine receptors from each of the chemokine families (C, CC, CXC, and CX3C), with the chemokine agonists along the wheel spokes (39, 44, 45). The colors assigned to the receptors are arbitrary and serve to distinguish the families visually. Chemokine receptors are generally classified as either homeostatic or inflammatory based on their predominant roles. However, this distinction is not absolute, as their activity and function depend strongly on the cellular and inflammatory context. In the figure, receptors associated with inflammation are shaded in red, those linked to homeostasis in blue, and receptors with dual functionality in yellow. Notably, other inflammatory receptors may also contribute to homeostatic processes, given their role in tissue repair, although this remains to be further explored. In the center, there are some leukocytes that express chemokine receptors. Created in BioRender; Proost, P (2025).

Currently, there are 20 identified conventional chemokine receptors, and they are widely expressed in leukocytes (**Figure 1**) (39, 44, 45). During inflammation, chemokines orchestrate leukocyte trafficking, guiding cells to sites of injury or infection. Although often discussed in simplified terms, most chemokine receptors exhibit pleiotropic and context-dependent functions that extend beyond mere recruitment. Briefly, CXCR1 and CXCR2 for instance, are predominantly associated with neutrophil recruitment and activation, yet they also participate in angiogenesis, epithelial regeneration, and tumor biology; CXCR3 is largely expressed on activated T cells and NK cells, where it mediates effector cell migration to inflamed tissues. However, the ligands of CXCR3 have been shown to downregulate angiogenic processes (46). CXCR4 plays multifaceted roles including progenitor cell retention in the bone marrow, T and B cell homing to secondary lymphoid organs, regulation of dendritic cell migration, and guidance of hematopoietic and endothelial precursors. Similarly, CXCR5 is critical for the positioning of B cells within germinal centers and for T follicular helper cell localization (34, 39).

CCR receptors are highly versatile and collectively govern the migration of monocytes, various lymphocyte subsets, NK cells, eosinophils, basophils, and dendritic cells. In particular, CCR2 and CCR7 are essential for monocyte egress from the bone marrow and dendritic cell trafficking to lymph nodes, respectively (28, 45). CX3CR1, on the other hand, mediates adhesion and migration of monocytes, macrophages, dendritic cells, T cells, and NK cells, contributing to both immune surveillance and tissue homeostasis (47).

As previously mentioned, chemokines and their receptors may have multiple functions or bind differently according to the context. Thus, it is important to notice that they can recruit other cells and can be involved in different processes (48). For instance, CCL3, CCL4, and CCL5 bind to CCR5 to recruit immune cells during inflammation, but also play a role in HIV suppression by competing with viral gp120 for binding to CCR5, resulting in reduction of viral entry in the target cells (49, 50). Similarly, CXCL12, which is essential for hematopoietic stem cell homing, is also exploited by tumor cells for metastasis and competes with HIV-1 gp120 for binding to CXCR4 (51). CXCL8, as main human neutrophil chemoattractant, directs neutrophil migration during acute inflammation, but also promotes tumor growth through stimulation of angiogenesis and epithelial-to-mesenchymal transition (52). As shown in **Figure 1**, chemokines may be associated with general inflammation or homeostasis, but are not commonly described as being implicated in the resolution of inflammation. Nevertheless, evidences indicate that both their presence and absence can affect immune cell accumulation and activation during resolution.

## Role of chemokines in resolution of inflammation

### Depletion of inflammatory chemokines

**Figure 2:**
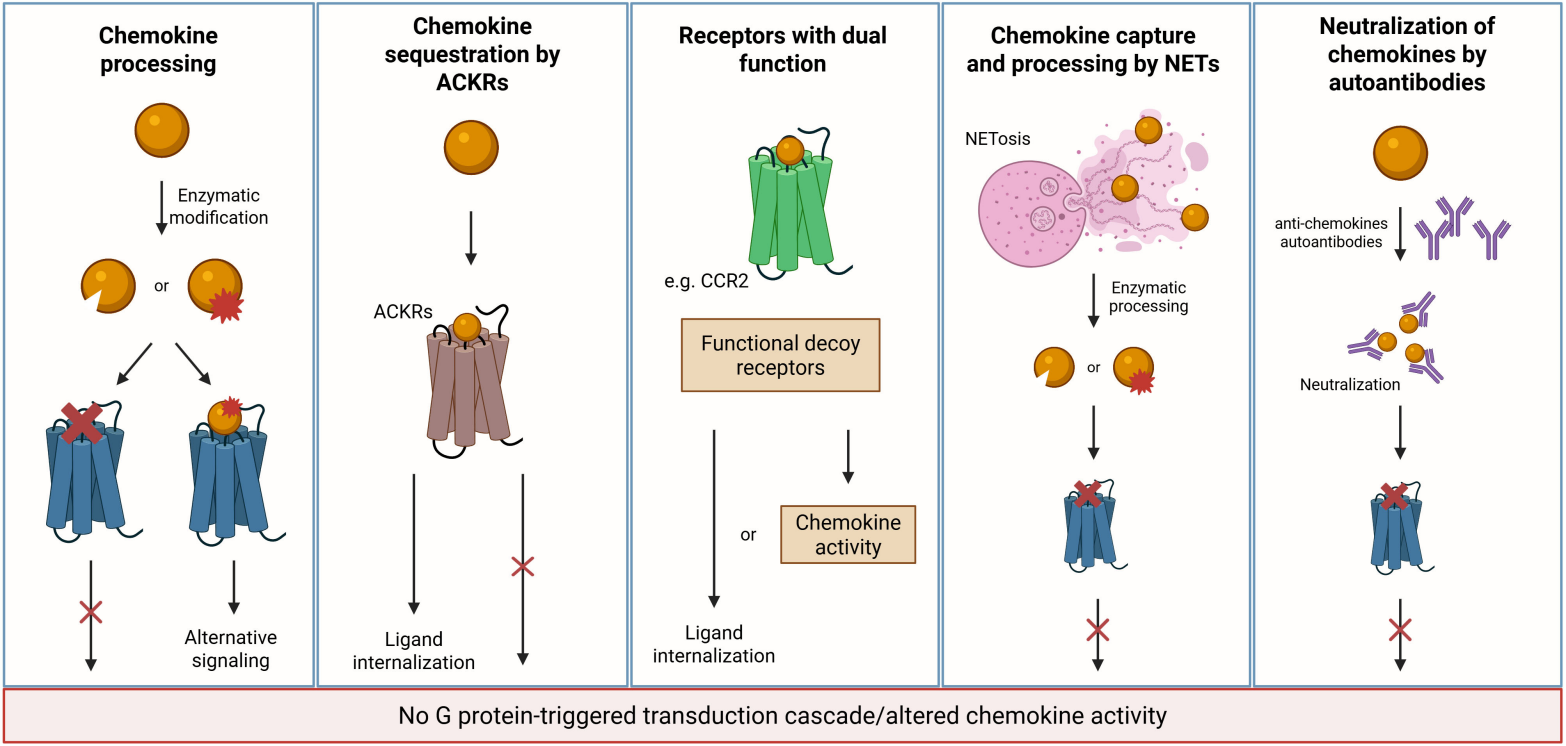
Mechanisms of chemokine depletion/inactivation during resolution. Multiple mechanisms contribute to the clearance or inactivation of chemokines as inflammation resolves. MMPs and other proteases cleave CC and CXC chemokines, generating inactive fragments or molecules with altered receptor specificity. ACKRs function as scavengers by internalizing chemokines without triggering G protein-coupled signaling but resulting in ligand internalization and degradation. Classical receptors may transiently adopt a decoy-like role under specific conditions. Specifically, CCR2 has been shown to act as a chemokine sink, internalizing excess CCL2 to limit inflammatory signaling and promote resolution. Additionally, NETs can capture and immobilize cytokines and chemokines, reducing their bioavailability and facilitating the transition toward resolution. Autoantibodies that neutralize chemokines can also contribute to their inactivation. Created in BioRender; Proost, P (2025).

One of the initial steps in resolving inflammation is to reduce neutrophil accumulation at the inflammatory site, mainly by abrogating their recruitment, but potentially also through stimulation of reverse migration (53). Therefore, the depletion of active inflammatory chemokines in the inflammation site is crucial to reduce the inflammatory response, facilitating tissue repair, and restoring homeostasis. Various mechanisms contribute to reducing levels of active chemokines, including enzymatic processing, capture by neutrophil extracellular traps (NETs), and sequestration by receptors without activation of G proteins (**Figure 2**). Besides depletion, recent studies have shown that chemokines are neutralized by autoantibodies, and this may modulate inflammation and promote its resolution (54, 55).

#### Depletion of inflammatory chemokines by enzymatic processing

Different enzymes can affect the function of chemokines, inducing several post-translational modifications such as site specific proteolytic cleavage, citrullination, nitration and glycosylation. In this context, proteolytic processing has been shown, primarily *in vitro*, to play a crucial role in the activity regulation of chemokines. Several enzymes can cleave chemokines, including matrix metalloproteinases (MMPs), dipeptidyl peptidase IV (DPP4/CD26), and aminopeptidases such as aminopeptidase N (ANP/CD13) (29). These modifications can significantly influence the inflammatory response, not only by inactivating chemokines but also by producing more potent chemokines, altering their receptor specificity or modifying their chemotactic properties (56).

MMPs are a large family of calcium-dependent, zinc-containing endopeptidases that were initially identified through their role in degradation of extracellular matrix components. However, they participate in various biological and physiological processes (57–61). MMPs can cleave chemokines at their N- or C-terminus, modulating their activity and altering leukocyte recruitment. For instance, macrophage-secreted MMP-12 cleaves CXC chemokines at the ELR motif, which is essential for receptor binding, abolishing the ability to recruit neutrophils, and illustrating how chemokine processing can be a pro-resolving mechanism (62). Beyond neutrophils, MMP-processing modifies the activity of several CC-chemokines, thereby influencing the recruitment of a broader range of leukocytes, including monocytes, macrophages, lymphocytes, dendritic cells, and eosinophils. For example, MMP-2 and MMP-9 cleave CCL2 and CCL7, leading to the loss of their chemotactic activity for monocytes and lymphocytes (63, 64). The truncated CCL7 form retains the capacity to bind CCR1, CCR2, and CCR3, but without inducing migration, thus serving as a functional antagonist that dampens inflammation (62, 65). Similarly, MMP-processing of other CC-chemokines, such as CCL8, CCL13, CCL15, and CCL23, has been shown to alter their ability to attract leukocytes (62, 64, 66), further contributing to the fine-tuning of cell trafficking. Notably, although CC chemokines are involved in the recruitment of various leukocyte subsets, most studies have predominantly focused on their role in monocyte attraction, highlighting a knowledge gap that remains to be addressed.

Besides MMPs, CD26/DPP4 is present as a membrane-bound serine protease on various immune and non-immune cells, and also in a soluble shed form in plasma. This specific enzyme removes the two most N-terminal amino acids from a broad list of chemokines, including CCL3L1, CCL4, CCL5, CCL11, CCL14, CCL22, CXCL2, CXCL6, CXCL9, CXCL10, CXCL11, and CXCL12 (56). CD26/DPP4-mediated cleavage of chemokines often significantly impacts their activity, either enhancing or diminishing chemokine activity or altering the receptor specificity depending on the substrate. While some chemokines such as CCL3L1 are activated by CD26/DPP4, CCL5 becomes a specific CCR5 agonist and others including CXCL10, CXCL12, CCL11, and CCL22 are inactivated following truncation (67). For instance, CXCL12 cleavage by CD26/DPP4 decreases its affinity for CXCR4, significantly reduces downstream calcium signaling and CXCL12-driven cell migration (68, 69).

Aminopeptidase N (APN)/CD13 is another membrane-bound protease with a wide range of functions, including chemokine processing (70). Together with CD26/DPP4, APN/CD13 generates truncated forms of CXCL11 with reduced binding, signaling, and chemotactic properties. This proteolytic process dampens the immune response (71) and, consequently, promotes tissue repair. In contrast, CXCL8 appears to be resistant to cleavage by APN/CD13, highlighting the complexity of chemokine-protease interactions. This resistance emphasizes the need to investigate chemokine - protease interactions in different biological contexts, as their effects may vary depending on the specific chemokine and the tissue environment (71, 72).

In addition to proteolytic cleavage, citrullination, nitration and glycosylation may affect chemokine activity. For instance, citrullination of CXCL12 reduces its biological function, with fully citrullinated CXCL12 being inactive (73). Similarly, citrullinated CXCL8 displays reduced chemoattractant and signaling capacity through CXCR2, as well as reduced affinity for GAGs (74). Similar functional impairments have been reported for CXCL10 and CXCL11, which, despite maintaining their ability to bind CXCR3, show reduced signaling activity following citrullination (75). Nitration also reduces the activity of CCL2 (76), CXCL8 (77) and CXCL12 (78). Although glycosylation has been reported on only a few chemokines (e.g. on CCL2, CCL14 and CX3CL1) and may affect protein stability (as evidenced for CCL14), limited information is available on its biological effects. Reports up to now rather point towards fine tuning rather than abolishing chemokine activity. For instance, CX3CL1 is a membrane-bound and highly glycosylated chemokine and the extend of its glycosylation regulates the presentation of the chemokine domain to the receptor CX3CR1 (79). In addition, evidence is accumulating for a regulatory role for glycosylation and tyrosine sulfation of a large number, if not almost all chemokine receptors (80–86). However, the fact that glycosylation is regulated by about 200 glycosyltransferase enzymes of which the expression differs between cells and alters depending on the activation of cells further complicates the study of the impact of glycosylation (87).

Importantly, the spatial and temporal expression and activity of these chemokine and chemokine receptor processing enzymes is tightly regulated, ensuring that chemokine activity is fine-tuned according to the inflammatory stage and tissue environment (88–92). Dysregulation of this processing system has been associated with chronic inflammatory diseases, such as chronic obstructive pulmonary disease (COPD) (93), cystic fibrosis (CF) (94), and rheumatoid arthritis (95). Furthermore, since a number of these enzymes also process cytokines, hormones, receptors, and/or extracellular matrix components, they participate in a broader regulatory network that coordinates immune responses and tissue remodeling.

#### Depletion of inflammatory chemokines by NETs

Another mechanism of chemokine depletion involves the release of NETs. These are net-like structures composed of DNA, histones and antimicrobial proteins. Notably, there are several enzymes associated to NETs that are released by activated neutrophils such as proteases and peptidylarginine deiminases that citrullinate proteins (96). These structures were first identified as a defense mechanism against pathogens, trapping and neutralizing bacteria, fungi, and viruses and facilitating their phagocytosis (97). It is known that recruited neutrophils release NETs to constrain the infection, but additionally to propagate the inflammation (98, 99). However, subsequent research has revealed that NETs also play a significant role in modulating inflammation. After the initial influx of neutrophils, the NETs aggregate, forming AggNETs, which sequester and degrade inflammatory mediators, dismantling chemokine and cytokine tissue gradients and inhibiting further neutrophil recruitment (100). This was demonstrated in a model of monosodium urate (MSU) crystal-induced gout, where NETs interrupt the inflammatory cycle by stimulating degradation of chemokines and proinflammatory cytokines, thus bringing recruitment of cells and NETosis to a halt and contributing to resolution of inflammation (101).

#### Depletion of inflammatory chemokines by decoy receptors

Chemokine receptors play a crucial role in regulating chemokine activity, and among them, atypical chemokine receptors (ACKRs) are especially important for fine-tuning chemokine availability. These seven-transmembrane spanning receptors differ from classical chemokine receptors in their broad ligand-binding profiles, and their inability to trigger conventional G protein-dependent signaling pathways (102, 103). Structurally and functionally diverse, the atypical chemokine receptor family includes five members: ACKR1, also called Duffy antigen receptor for chemokines (DARC); ACKR2, also called D6; ACKR3, also called CXCR7; ACKR4, previously called CCRL1 or Chemocentryx chemokine receptor (CCX-CKR); and ACKR5, also called GPR182 (36). These receptors have different roles and are mostly known to bind and constitutively internalize their ligands. Acting like decoy receptors, the ACKRs lack the DRY motif in the second intracellular loop, which is involved in coupling to G proteins and is essential to initiate these classical signaling cascades. While the receptor is recycled back to the plasma membrane, the ligand is directed toward lysosomal degradation. Notably, this internalization and recycling may occur independently of ligand presence. It is known that the absence of ACKRs leads to dysregulated accumulation of leukocytes and has different consequences (104, 105).

For instance, the absence of ACKR2 exacerbates inflammatory responses in several murine models. In the skin, ACKR2-deficient mice exhibit heightened inflammation following phorbol ester administration or complete Freund’s adjuvant (CFA) injection (105, 106). Similarly, these mice show increased sensitivity to carbon tetrachloride-induced acute liver injury (107). In lung models, the absence of ACKR2 leads to increased recruitment of eosinophils and dendritic cells in allergic asthma (108), and, after *Mycobacterium tuberculosis infection*, results in abnormal leukocyte accumulation in the lungs, kidneys, liver, and lymph nodes, eventually decreasing survival (109). In human disease, ACKR2 expression is found to be elevated in unaffected skin regions of patients with psoriasis (110) and in alveolar macrophages of individuals with chronic obstructive pulmonary disease (111), potentially due to a compensatory mechanism to excessive leukocyte recruitment (112). In another context, the lack of ACKR3 in murine embryos results in an increased number of natural killer and dendritic cells in the placenta, which in turn leads to an abnormal cytotoxic response and inefficient trophoblast invasion (113). Interestingly, reduced expression of ACKR3 has been observed in trophoblast cells from pregnancies affected by preeclampsia, suggesting a critical role for this receptor in placental development (114).

In addition to structural decoy receptors, functional decoy receptors have been identified within the chemokine system (115, 116). These receptors are structurally identical to signaling chemokine receptors. However, under specific environmental conditions, they become uncoupled from intracellular signaling pathways and do not induce cellular activation or migration. Nonetheless, they still bind and sequester their ligands, effectively functioning as decoy receptors. The expression of chemokine receptors that naturally fail to trigger intracellular signaling has long been observed in several cell types, such as monocytes and dendritic cells (117). This is observed when the cells are simultaneously exposed to a classical inflammatory signal such as lipopolysaccharide (LPS) together with the anti-inflammatory cytokine IL-10. Under these conditions, IL-10 prevents the typical downregulation of CCR1, CCR2, and CCR5 that LPS induces in monocytes and dendritic cells. Despite high surface expression of these receptors, the cells are unable to migrate in response to chemokines such as CCL2, CCL3, CCL4, or CCL5 (116). Besides the silencing, the expression of these receptors can be increased in apoptotic neutrophils to sequester more chemokines and lead to chemokine depletion and resolution of inflammation (118). Notably, recent findings revealed that CCR2 can act as both a signaling and scavenging receptor, highlighting that even classical chemokine receptors may dynamically switch between these functions depending on the cellular context and inflammatory environment (119).

#### Neutralization of inflammatory chemokines by autoantibodies

Emerging evidence suggests that autoantibodies targeting chemokines may assist in the resolution of inflammation. These naturally occurring or infection-induced antibodies can selectively neutralize pro-inflammatory chemokines, thereby reducing leukocyte recruitment and dampening excessive immune activation. Elevated levels of such neutralizing antibodies have been observed in individuals recovering from COVID-19, where higher titers correlated with milder disease and faster recovery, supporting their protective, inflammation-limiting function (55, 120). In cancer and autoimmune contexts, they are increasingly recognized as endogenous regulators of inflammation, potentially contributing to immune homeostasis and preventing chronic inflammatory responses (54, 121, 122). Collectively, these findings reveal an additional regulatory layer within the chemokine network, suggesting that the adaptive immune system itself can aid in resolving inflammation through the generation of chemokine-specific autoantibodies.

### Reverse migration

Besides the classical cell migration, chemokines may be involved in regulating the reverse migration of neutrophils, and this might be important for resolving inflammation. Neutrophils are typically the first responders recruited to an inflammatory site, and their prompt clearance from the tissue is vital to prevent excessive tissue damage. Although the apoptosis of neutrophils is considered a hallmark of resolution of inflammation, some studies have shown that neutrophils at the site of the inflammation may return to the main circulation and do not undergo apoptosis locally (53, 123, 124).

In the reverse migration process, the plasma levels of certain chemokines, such as CXCL1 and CXCL2, or other chemokine receptor ligands, are increased, creating a gradient from the inflammation site to the blood circulation, in other words, the classical chemotactic gradient is inverted. In turn, the endothelial cells capture these chemokines and present them to neutrophils in the inflammation site, inducing the reverse migration (125–127). It has been show that, in a murine air pouch model, neutrophils that regress to the blood vessels have increased expression of CCRL2 and the level of chemerin, a CCRL2 ligand, in plasma is increased in late stages of inflammation. In the same study, the neutralization of chemerin led to a reduction in reverse migration, suggesting that circulating chemerin attracts neutrophils to leave inflammatory sites by interacting with CCRL2 (128). In zebrafish, reverse migration has been shown to depend on CXCL8a and its receptor CXCR2. Within this model, CXCR2 regulates neutrophil behavior in inflamed tissues and contributes to the resolution of inflammation by limiting neutrophil accumulation at the wound site (129). After returning to the blood vessels, neutrophils can stay in circulation as a distinct population of long-lived cells, as observed in peripheral blood of patients with chronic inflammatory arthritis and atherosclerosis (130). Alternatively, neutrophils can return to the bone marrow (126) or migrate to remote organs, such as lungs, where they may have pathological effects (127). These findings highlight the dual nature of reverse migration: while it may contribute to the resolution of local inflammation, it might also lead to inflammatory responses in remote tissues. Despite its importance in experimental models, the clinical relevance of reverse migration in humans remains under investigation, as most of the current understanding is derived from murine and zebrafish studies (127–129, 131).

Together with the depletion of chemokines and the clearance of apoptotic cells by macrophages, the increase of chemokines in the blood circulation in late stages of inflammation leads to the reduction of local neutrophil accumulation and allows the return of homeostasis. Further studies are needed to explore the consequences of reverse migration and potential strategies to manipulate this process, in which the chemokine system plays a key role.

### Recruitment and polarization of pro-resolving cells

The recruitment of pro-resolving leukocytes by chemokines is an essential step for transitioning from inflammation to tissue repair and homeostasis. This recruitment involves a shift in the type of cells present in the tissue, replacing pro-inflammatory cells with pro-resolving ones, such as pro-resolving macrophages, Tregs, ILCs, and others (1, 26). Certain chemokines, such as CCL2, CCL5, and CXCL12, are known to attract monocytes and macrophages to sites of inflammation, where they can contribute to the resolution process by clearing apoptotic cells and debris (132). Similarly, CCR4 and its ligands, CCL17 and CCL22, are essential for the recruitment of Tregs, that contribute to immune suppression by releasing anti-inflammatory cytokines such as IL-10 and by directly interacting with other immune cells to dampen their activity (133–137). Interestingly, engulfment of apoptotic cells by CD169^+^ macrophages induces expression of CCL22 that, in turn, recruits Tregs via CCR4, showing once more how efferocytosis triggers several events linked to resolution (138). In addition to CCR4, Treg migration is regulated by several other chemokine receptors, including CCR7, which guides Tregs to lymph nodes in response to CCL19 and CCL21 (139), and CCR5, which facilitates their homing to inflamed tissues via CCL3 and CCL4 (140). CXCR3 has also been implicated in directing Tregs to sites of Th1-type inflammation through CXCL9, CXCL10, and CXCL11 (141), while CCR8 and its ligand CCL1 have been shown to mediate Treg accumulation in tumor microenvironments (142).

In contrast, CCR2 plays a major role in monocyte recruitment but shows context-dependent roles. For instance, the recruitment of monocytes, or rather its reduction, does not affect the resolution of acute respiratory distress syndrome (ARDS) induced by either malaria (143) or LPS (144) in mice. Although the absence of monocytes does not directly affect disease progression or resolution, likely due to compensatory mechanisms such as alveolar macrophage proliferation (144), the lack of CCR2 might disrupt the restoration of homeostasis, since it impairs the reappearance of eosinophils and interstitial macrophages during recovery (143). Nonetheless, in a model of lung injury induced by infection with influenza A or treatment with bleomycin, as well as in a model of lung injury induced by acetaminophen, a subpopulation of macrophages, dependent on CCR2, was discovered and associated with tissue repair and regeneration (145). In a context of wound healing, CCR2 has also been described as essential given that, once the CCR2/CCL2 axis is disrupted by stimulator of interferon genes (STING) deficiency, the recruitment of monocytes and the accumulation of macrophages in the wound is significantly reduced. In other words, CCR2/CCL2 signaling, in particular, facilitates the recruitment of Ly6C^high^ monocytes from the bone marrow and their migration to the wound site, which is essential for replacing neutrophils and promoting wound healing. In the absence of proper chemokine signaling, monocyte recruitment is impaired, leading to prolonged inflammation and delayed resolution (146).

The effects of chemokines on macrophages, however, goes beyond their recruitment. They also participate in the regulation of apoptosis and recognition and clearance of apoptotic cells. CCL1 signaling through its receptor CCR8 on lymphocytes inhibits apoptosis (30). On the other hand, CX3CL1 (fractalkine) functions as a *“find-me”* signal released by apoptotic cells, attracting phagocytes such as macrophages to the site of cell death and facilitating the initiation of efferocytosis (147). Also in hepatic ischemia-reperfusion injury models, the CX3CL1/CX3CR1 axis plays a regulatory role in macrophage-mediated recovery. Specifically, CX3CR1 signaling modulates the expression of MerTK, a receptor tyrosine kinase essential for efferocytosis. Interestingly, CX3CR1 deficiency enhances the capacity of macrophages to clear apoptotic cells and leads to compensatory upregulation of other chemokine receptors such as CCR1 and CCR5. This results in increased immigration of monocytes, that differentiate locally to macrophages to replenish the Kupffer cell population and contribute to tissue homeostasis (148).

Besides recruitment and modulation of macrophage activity, chemokines play a key role in guiding macrophage polarization toward either the pro-inflammatory M1 or the pro-resolving M2 phenotype (149). For instance, Li et al. (150) have demonstrated that knocking down CCR1 or CCR5 can impair CCL5-induced MAPK and NF-κB activation. This, in turn, decreases M1 polarization while simultaneously enhancing IL-4-induced M2 polarization. On the other hand, certain chemokines facilitate the polarization of macrophages into the M2 phenotype, which is associated with anti-inflammatory responses and tissue repair. Specifically, CCL22 has been shown to induce the polarization of tumor-associated macrophages into the M2 subtype in the context of cervical cancer. This process may significantly contribute to progression and increased aggressiveness of a tumor (151, 152). Also in the context of cancer, CXCL13 has been implicated in promoting M2 macrophage polarization and facilitating tumor progression in myeloma osteolytic disease. Similarly, but in other circumstances, CCL24 plays a fundamental role in angiotensin II-induced heart failure by promoting the polarization of macrophages toward the M2 phenotype (152). Taken together, these data indicate that chemokines play an important and multifaceted role in the context of the localization of specific subpopulations of macrophages, as they are not only involved in the selective recruitment of cells but also actively contribute to their polarization.

It is important to recognize that, while chemokines are considered important co-factors, they are not the primary drivers of macrophage polarization. Cytokines such as interferon (IFN)-γ and IL-4/IL-13 are well-established as crucial regulators in this process (23, 153). Thus, the interplay between chemokines and the production of these cytokine mediators must be taken into consideration when evaluating macrophage polarization.

In addition to macrophages, neutrophils have increasingly been recognized for their pro-resolving functions. The most studied role of neutrophils in resolution is that they become apoptotic and are cleared by macrophages, leading to a cascade of anti-inflammatory and tissue-repair responses. However, as already discussed, neutrophils contribute to resolution through mechanisms beyond their own death (154, 155). These cells may participate in dampening complement activation and inflammation (3), promote cytokine scavenging (101), remove tissue debris (156), induce matrix remodeling (157), and support angiogenesis and tissue regeneration (158, 159).

Although several studies highlight neutrophil phenotypic heterogeneity and functional plasticity, the link between different phenotypes and specific neutrophil subpopulations is not completely understood, particularly outside the context of cancer. Currently, neutrophils may be classified into N1, with pro-inflammatory or anti-tumor functions, and N2, with pro-resolving or pro-tumor functions. For instance, the number of N2 neutrophils is increased after myocardial infarction which may help to prevent further wall thinning of the ventricle (160). Moreover, a study about resolution of liver injury has shown that, following the phagocytosis of cellular debris, neutrophils underwent a shift in gene expression towards an anti-inflammatory and pro-resolving profile. This includes the upregulation of receptors such as CXCR2 and CXCR4, which mediate reverse migration and homing to the bone marrow, as well as the expression of anti-inflammatory and pro-resolving mediators such as IL-10 and annexin A1 (ANXA1) (161). Similarly, a pro-resolving population of neutrophils has been identified in late stages of mouse acute liver injury (162). This population was characterized by the downregulation of pro-inflammatory genes and is associated with modulation of macrophage activity and polarization, as well as promotion of angiogenesis. CXCR4, whose expression in neutrophils has been linked to angiogenesis and tissue repair, is among the surface markers of this subset (163, 164). In contrast, the pro-inflammatory neutrophil subpopulation expresses CXCR5, highlighting how the expression of specific chemokine receptors plays a pivotal role in guiding the recruitment of functionally distinct neutrophil subsets to the liver (162). Interestingly, another study has shown that the activation of the CCL20-CCR6 axis via TNF-α recruits proangiogenic VEGFA^+^ neutrophils to sites of ischemic injury leading to initiation of angiogenesis and tissue regeneration (165). More about the different subtypes of neutrophils *in vivo* and the role of the chemokine system in this process is yet to be discovered.

### Promotion of disease recovery and tissue repair

Chemokines are traditionally known for their role in initiating and amplifying inflammatory responses, but growing evidence highlights their importance in resolving inflammation and promoting recovery across various pathological contexts. Once the inflammatory phase subsides, chemokines contribute to the restoration of tissue homeostasis by orchestrating the clearance of apoptotic cells, recruiting reparative leukocytes, and directing stromal, epithelial, and progenitor cells to damaged areas. This spatiotemporal regulation ensures that immune responses are terminated efficiently, preventing chronic inflammation and fibrosis.

In COVID-19, for instance, chemokines and their receptors modulate the mucosal immune response, promoting viral clearance while facilitating symptom resolution. According to Cass et al. (166), increased levels of the chemokines CCL13, CCL17, and CCL26 are associated with the development of a Th2-like immune response in the upper respiratory tract. These chemokines recruit and activate key immune players such as ILCs, B cells, and eosinophils, which contribute to virus control and tissue integrity recovery. Interestingly, therapeutic intervention with budesonide, a corticosteroid commonly used for asthma and early COVID-19 treatment (167), can enhance the expression of CCL17. This upregulation was associated with improved clinical outcomes, without a demonstrable direct antiviral effect (166).

Aberrant chemokine signaling can, however, impair resolution and delay healing. In WHIM (warts, hypogammaglobulinemia, infections, and myelokathexis) syndrome, gain-of-function mutations in CXCR4 prevent receptor desensitization, leading to immune cell retention in the bone marrow and contributing to osteopenia and defective tissue regeneration (168). In this context, diminishing CXCR4 activity is beneficial (169). Mice treated with plerixafor (AMD3100), a CXCR4 antagonist, showed a restored osteogenic capacity, highlighting the importance of a proper balance in the chemokine system (170, 171).

Besides leukocytes, chemokines regulate the migration of fibroblasts and endothelial cells, both of which are crucial for wound healing and tissue remodeling. Importantly, angiogenesis and lymphangiogenesis are not restricted to the resolution phase. They also occur during inflammation, where they contribute to leukocyte recruitment and nutrient supply to inflamed tissues (172). However, as inflammation resolves, these same processes become essential for tissue repair and restoration of homeostasis (173).

Chemokines such as CCL2 and CXCL8, as well as other CXCR2 ligands, recruit fibroblasts and endothelial cells to sites of injury, promoting extracellular matrix (ECM) production and angiogenesis (174–176). CCR7 and its ligands CCL19 and CCL21 also participate in fibroblast recruitment in inflammatory conditions such as rheumatoid arthritis (177) and idiopathic pulmonary fibrosis (178). These fibroblasts display enhanced migration and proliferation capacities and secrete vascular endothelial growth factor (VEGF), which further promotes angiogenesis (177). Beyond VEGF secretion, the CCR7/CCL21 axis influences macrophage polarization and Th17 cell differentiation, leading to osteoclast activation and subsequent vascular remodeling (179). Similarly, in skin wounds infected with *Enterococcus faecalis*, CXCL2 acts together with SPP1 (osteopontin) to coordinate pathogen control and tissue regeneration. In this context, CXCL2 not only recruits neutrophils to limit infection but also promotes angiogenesis, highlighting the dual role of certain chemokines in both inflammation and tissue repair (180, 181).

Another important chemokine in the angiogenesis context is CXCL12. Although VEGF is the most studied and recognized factor for migration and proliferation of endothelial cells (182), CXCL12 coordinates the migration of endothelial cells through the spatially restricted activation of ACKR3 and CXCR4b (183), promoting angiogenesis. In ischemic injury, CXCL12-CXCR4 signaling promotes endothelial progenitor cell (EPC) recruitment and vascular regeneration, facilitating angiogenesis and restoring perfusion (184). Furthermore, CXCL12 is essential for promoting tissue regeneration in organs such as the lungs and liver by enhancing cell survival, proliferation, and differentiation (185, 186). Although CXCL12 classically binds CXCR4, it also interacts with ACKR3 that is involved in angiogenesis and cell proliferation (187, 188). Treatment with ACKR3 antagonists suppressed symptoms of collagen-induced arthritis in DBA/1 mice, reducing the number of blood vessels in inflamed joints. These findings suggest that disease suppression may be mediated, at least in part, by the inhibition of pathological angiogenesis. Consistently, strong expression of ACKR3 has been found on endothelial cells in the synovium of rheumatoid arthritis patients, suggesting a similar mechanism in human disease (112, 189).

Chemokines also promote lymphangiogenesis, a process essential during both inflammation and resolution. In inflamed tissues, lymphangiogenesis facilitates the drainage of edema and the trafficking of immune cells, whereas in the resolution phase, it contributes to clearing interstitial fluid, apoptotic cells, and inflammatory mediators. CCL21, via its receptor CCR7, promotes the formation of new lymphatic vessels, an essential step in resolving tissue edema and removing cellular debris (190). This has been demonstrated in patients with idiopathic diffuse alveolar damage, in which CCR7^+^ macrophages were found around the newly formed lymphatics, suggesting they may participate in lymphangiogenesis, facilitated by CCL19 from the lymphatic endothelium (191). This contributes to immune homeostasis and ensures that the inflammatory response resolves efficiently, preventing further tissue damage (173). Further along the resolution process, chemokines regulate the migration, retention, and activation of progenitor and stem cells, which are crucial for tissue regeneration and remodeling. For instance, CXCL12 plays a central role in maintaining hematopoietic stem and progenitor cells (HSPCs) in the bone marrow niche via CXCR4 signaling, and its gradient guides the mobilization of these cells into peripheral tissues during repair (192, 193). Moreover, CCL11 regulates the trafficking of neural progenitor cells in models of brain injury, suggesting that chemokine-mediated progenitor mobilization extends beyond classical immune repair mechanisms (194).

In addition to the many chemokines implicated in angiogenesis and lymphangiogenesis, CCL5 produced by pro-resolving macrophages appears to contribute to tissue repair by promoting wound healing (195). This effect appears to be partially mediated through the recruitment of stromal cells via CCR1 (196). Other chemokines, such as CXCL12 and CCL2, play crucial roles by promoting stromal cell homing, proliferation, and differentiation within damaged tissues, thereby supporting angiogenesis and tissue remodeling (197, 198).

Importantly, the ability of chemokines to promote tissue repair appears to depend on the surrounding microenvironment. When acting alongside anti-inflammatory mediators, chemokines may support tissue regeneration and recovery, whereas their association with pro-inflammatory factors can hinder healing. In line with this, a pro-resolving macrophage secretome rich in anti-inflammatory cytokines and chemokines such as CCL5, CXCL2, and CCL22 has shown therapeutic efficacy in experimental models of inflammation (199–201). Conversely, increased CCL5 production together with type IFN-I has been associated with defective mucosal repair in Crohn’s disease (202).

## The chemokine system as a therapeutic target

Several chemokines and their receptors are under investigation as therapeutic targets (203). In the context of cancer, the chemokine receptors CCR4 and CXCR4 have been extensively investigated. An anti-CCR4 monoclonal antibody, mogamulizumab, has been approved for the treatment of mycosis fungoides and Sézary syndrome, and is under evaluation for use in other types of cancer (204–206). Given that CCR4 is highly expressed in effector Tregs but not in naïve Tregs, mogamulizumab selectively depletes effector Tregs without affecting naïve Tregs, which remain essential for autoimmune suppression (207). Due to its overexpression in multiple cancer types and its well-established role in tumor progression via the CXCR4/CXC12 axis, CXCR4 is another therapeutic target, with inhibitors such as plerixafor being used for autologous transplantation of bone marrow in patients with Non-Hodgkin’s lymphoma or multiple myeloma and as a chemosensitizer (208, 209). Also other antagonists of CXCR4 are being explored in this context. Among them, the CXCR4 antagonist mavorixafor enhances CD8^+^ cell infiltration and decreases immunosuppressive cells in the tumor microenvironment (210).

In 2024 the FDA approved mavorixafor for patients from 12 years of age on with WHIM syndrome to increase the number of circulating mature neutrophils and lymphocytes, highlighting the clinical potential of chemokine receptor targeting beyond oncology (211). Additionally, CXCR4 blockade with plerixafor has shown promising results in controlling osteoporosis in WHIM syndrome, although these studies are still at the preclinical stage (170, 171). Furthermore, drugs targeting CCR5, such as maraviroc, have been approved for the treatment of HIV infection, demonstrating how chemokine receptor blockade can be translated into effective therapies for infectious diseases.

Regarding the resolution of inflammation, inhibitors of chemokine receptors are being developed to limit the recruitment of inflammatory cells to affected tissues in diseases such as rheumatoid arthritis, multiple sclerosis, and inflammatory bowel disease. For instance, CCR5 and CCR2 antagonists are being evaluated for their potential to reduce excessive immune cell infiltration and promote Treg cell generation in inflammatory conditions (212, 213). Moreover, given that chemokines play a role in tissue regeneration, modulating these pathways could promote repair of damaged tissue or prevent fibrosis (214). More specifically, the modulation of the CXCL12-CXCR4 axis is being vastly explored (52). Enhancing the effects of CXCL12 to promote stem cell differentiation and revascularization could be beneficial for organ repair and wound healing (215, 216). Conversely, in pathological conditions where angiogenesis is harmful, such as retinopathies and cancers, targeting and blocking CXCL12-CXCR4 signaling may have therapeutic advantages (217–219). Thus, the development of strategies aimed at either promoting or inhibiting CXCL12-driven pathways holds significant therapeutic potential but needs to be tailored to the specific context of interest (220). For instance, blocking CXCR4 with plerixafor has demonstrated pro-healing effects related to stem cell mobilization. In diabetic mice, a single topical application of plerixafor significantly accelerated wound healing by mobilizing bone marrow–derived endothelial progenitor cells and promoting fibroblast migration and proliferation (221).

Given its crucial role in maintaining homeostasis, various approaches are being explored to optimize the therapeutic use of CXCL12 while minimizing side effects, such as utilizing local administration to precisely target specific tissues (186, 216). In addition to directly targeting chemokine signaling, modulating chemokine degradation may also offer therapeutic opportunities, and both approaches can be combined, as demonstrated by Vågesjö and colleagues (222). In their study, the authors employed a CXCL12-delivering *Lactobacillus* strain that simultaneously lowered the local pH, thereby inhibiting CD26/DPP4, while producing CXCL12. This strategy significantly accelerated wound healing and the bacteria are currently in advanced stages of clinical testing as a therapeutic approach (223, 224). Furthermore, CD26/DPP4 is implicated in various inflammatory diseases due to its role in cleaving several chemokines. Therefore, inhibiting this enzyme could extend the lifespan of chemokines and, consequently, enhance their activity (225–227). In turn, the increase of chemokine activity might recruit specific cells with pro-resolving effects, such as Tregs (141). Nonetheless, despite its promise, modulation of CD26/DPP4 activity should be approached with caution, as this enzyme also processes neuropeptides, peptide hormones, vasoactive peptides, growth factors, and various cytokines (67).

Another promising therapeutic approach targeting the chemokine system is the use of glycosaminoglycan (GAG)-binding molecules. GAGs play a crucial role in the recruitment of immune cells by retaining chemokines on the endothelium and presenting them to circulating leukocytes, thereby facilitating the interaction between chemokines and their respective receptors (228, 229). Recent studies have highlighted the potential of GAG-binding peptides derived from chemokines, such as CXCL9 (74–103), as a therapeutic intervention. In murine models of inflammatory diseases, such as COVID-19 and antigen-induced arthritis, the administration of these peptides has been shown to reduce leukocyte recruitment to the sites of inflammation, likely due to the impairment of GAG-chemokine interactions (230–233). This reduction prevents excessive inflammatory responses and, as a result, tissue damage is decreased. While the specific impact on resolution of inflammation requires further investigation, it is reasonable to suggest that better regulation of leukocyte recruitment may contribute to a more effective resolution of inflammation.

**Figure 3:**
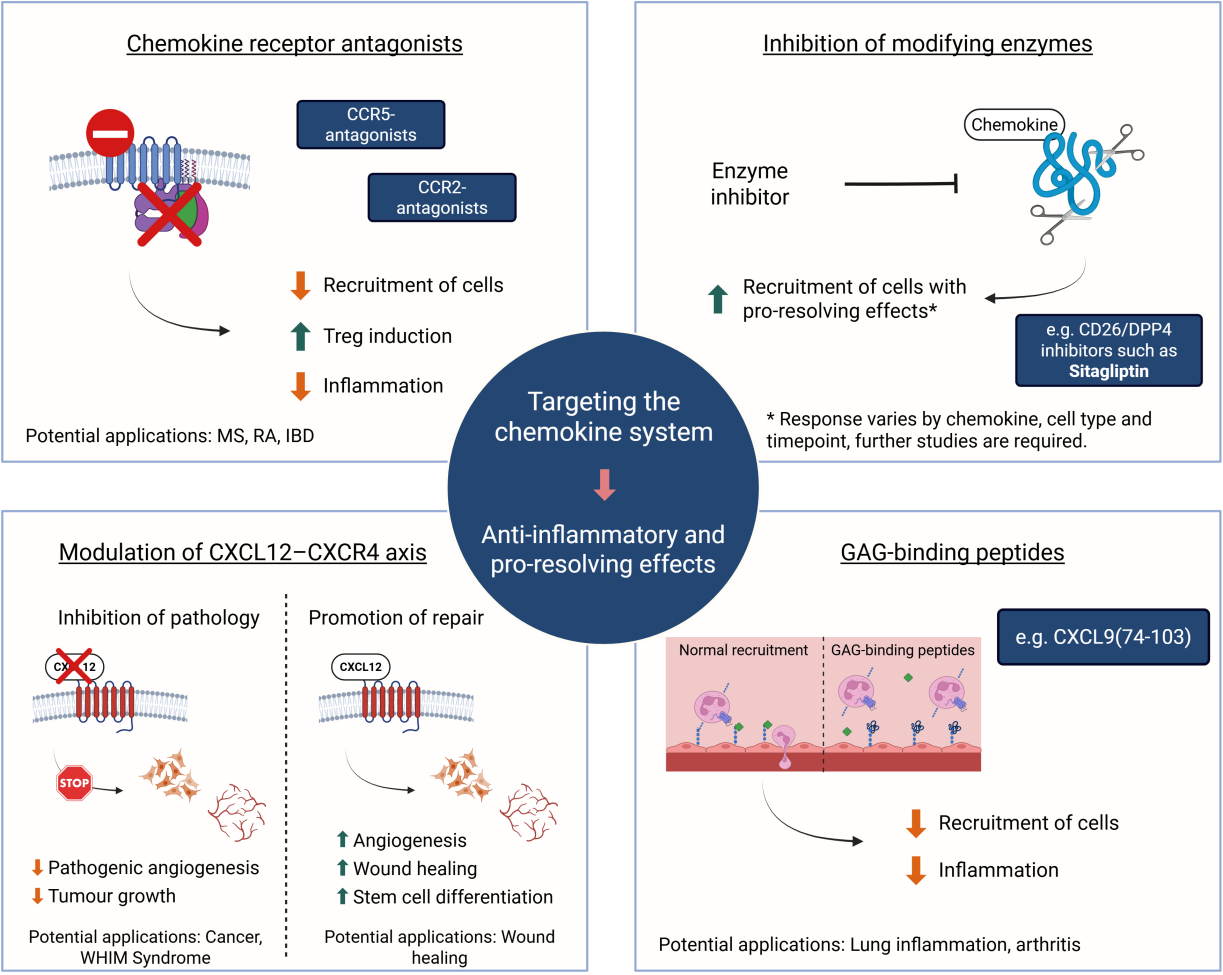
The chemokine system as a therapeutic target. Overview of current and emerging therapeutic strategies aimed at modulating chemokine activity. These include antagonizing chemokine receptors (e.g., CCR2, CCR5). Fine-tuning the CXCL12-CXCR4 axis may involve use of CXCR4 antagonists to reduce angiogenesis and tumor growth or to counteract gain-of-function mutations in WHIM syndrome. Alternatively, retaining CXCL12 signaling results in faster wound healing. Inhibiting the activity of chemokine modifying enzymes, such as DPP4/CD26, may stimulate recruitment of pro-resolving cells and disrupting interactions of inflammatory chemokines with GAGs through competition with potent GAG-binding peptides showed beneficial effects in preclinical models of lung and joint inflammation. Such interventions can modulate immune cell infiltration, promote inflammation resolution, and enhance tissue repair. The clinical outcomes of these strategies are highly context-dependent, varying with disease type, affected tissue, and timing of the intervention. Created in BioRender; Proost, P (2025).

Targeting the chemokine system is a promising strategy for both dampening inflammation and promoting resolution and tissue repair. Different approaches (**Figure 3**) have shown positive results in preclinical models. However, these outcomes are highly dependent on the specific context, including the tissue, disease stage, immune cell population, and timing of intervention. Despite the significant potential of molecules that target the chemokine network, the underlying mechanisms of this network and the effects of inhibiting or stimulating one member are not fully understood, and the therapeutic window remains to be explored. Much is yet to be discovered to ensure that chemokine-targeting therapeutic strategies are both effective and safe across different pathological settings.

## Future directions

The chemokine system has emerged as a critical regulator not only of immune cell recruitment during inflammation but also of its resolution. In addition, chemokine receptors, being GPCRs, make chemokines and their receptors therapeutic targets (203). While most current strategies focus on inhibiting pro-inflammatory chemokine signaling to limit immune cell infiltration, future research must deepen our understanding of how chemokines contribute to the later phases of inflammation, particularly in driving its resolution and promoting tissue repair.

One promising direction lies in the selective modulation of chemokine pathways that actively promote resolution, such as those involved in neutrophil reverse migration, macrophage polarization, and recruitment of regulatory and tissue-repairing cells. Future interventions in the chemokine system may focus on modulating the activity or availability of chemokines also by targeting enzymes responsible for posttranslational modifications. Similarly, precise temporal control over chemokine inhibition or enhancement, targeting specific phases of the inflammatory response, could minimize tissue damage while preserving essential immune defense mechanisms. The CXCL12-CXCR4 axis exemplifies this dual potential, where both the enhancement and inhibition of signaling may yield therapeutic benefits depending on the timing and the specific disease context (186).

Moreover, targeting the interactions between chemokines and GAGs is an emerging strategy with potential to regulate immune cell recruitment and gradient formation without directly interfering with receptor signaling (230, 232). As studies already have shown how GAG-binding peptides can modulate leukocyte recruitment, further investigation is needed to clarify their role in supporting inflammation resolution and limiting fibrosis.

Ultimately, translating chemokine-targeting therapies into effective clinical interventions requires a more nuanced understanding of their context-dependent roles in inflammation and the chemokine system redundancy. Integrating single-cell and spatial transcriptomics with functional studies may help identify precise chemokine signatures associated with resolution and repair across tissues and disease models. In parallel, expanding our knowledge on chemokine modifications, such as proteolytic modification, citrullination, glycosylation and nitration, and how they affect biological function may reveal new therapeutic opportunities, especially in the context of resolution (74, 77).

In summary, the next generation of chemokine-modulating therapies should move beyond inflammation suppression toward the fine-tuned orchestration of resolution. Exploring the full spectrum of chemokine functions and other molecules associated with that holds great promise for restoring immune balance and promoting recovery in inflammatory diseases.

## Concluding remarks

The chemokine system plays a critical role in both the inflammatory and resolution phases. During inflammation, chemokines mediate cellular recruitment to inflammation sites, and later, during resolution, chemokine depletion is the first step for limiting inflammation (28). Proteolytic processing by enzymes such as MMPs and CD26/DPP4, and the activity of atypical chemokine receptors, play a major role in modulating chemokine levels and activity (62, 234). However, chemokines appear to have a more complex role in resolving the inflammatory response than previously understood.

One of the mechanisms through which chemokines promote the resolution of inflammation is by facilitating the recruitment and polarization of pro-resolving cells, such as M2 macrophages and Tregs (149). These cells play critical roles in clearing apoptotic cells and dampening the immune response, thereby contributing to the restoration of tissue homeostasis and the resolution of inflammation. Furthermore, certain chemokines, such as CXCL1 and CXCL2, are implicated in reverse migration of neutrophils back to the circulation, thus preventing excessive tissue damage (53, 125, 126). Finally, chemokines are also important for tissue repair by recruiting fibroblasts, endothelial cells, and progenitor cells to damaged tissue. This facilitates extracellular matrix production, angiogenesis, and tissue regeneration, ensuring effective recovery. Chemokines such as CXCL12 and CCL21 are particularly important for stem cell recruitment and lymphangiogenesis, highlighting their complex role in inflammation and homeostasis (186, 190, 220).

In conclusion, the chemokine system plays a crucial dual role in both promoting and resolving inflammation by modulating cell recruitment, polarization, and tissue repair. Regarding the clinical implications, there are several options for therapeutically targeting the chemokine system and some are already being explored (203). Nevertheless, despite its vast potential, challenges remain in terms of specificity and balancing immune modulation without compromising host defense mechanisms. Future research should focus on further elucidating the precise mechanisms by which chemokines regulate the resolution of inflammation, possibly leading to new therapeutic strategies for controlling inflammation and enhancing tissue repair.
